# ASAP Score *versus* GALAD Score for detection of hepatitis C-related hepatocellular carcinoma: A multicenter case-control analysis

**DOI:** 10.3389/fonc.2022.1018396

**Published:** 2022-09-30

**Authors:** Si-Yu Liu, Chao Li, Li-Yang Sun, Ming-Cheng Guan, Li-Hui Gu, Dong-Xu Yin, Lan-Qing Yao, Lei Liang, Ming-Da Wang, Hao Xing, Hong Zhu, Timothy M. Pawlik, Wan Yee Lau, Feng Shen, Xiang-Min Tong, Tian Yang

**Affiliations:** ^1^ Department of Laboratory Medicine, Lishui Municipal Central Hospital, the Fifth Affiliated Hospital of Wenzhou Medical University, Lishui, China; ^2^ The Key Laboratory of Tumor Molecular Diagnosis and Individualized Medicine of Zhejiang Province, Zhejiang Provincial People’s Hospital, People’s Hospital of Hangzhou Medical College, Hangzhou, China; ^3^ Department of Hepatobiliary Surgery, Eastern Hepatobiliary Surgery Hospital, Second Military Medical University (Navy Medical University), Shanghai, China; ^4^ Department of Hepatobiliary, Pancreatic and Minimal Invasive Surgery, Zhejiang Provincial People’s Hospital, People’s Hospital of Hangzhou Medical College, Zhejiang, China; ^5^ Department of Medical Oncology, the First Affiliated Hospital of Soochow University, Suzhou, China; ^6^ School of Clinical Medicine, Hangzhou Medical College, Hangzhou, China; ^7^ Eastern Hepatobiliary Clinical Research Institute (EHCRI), Third Affiliated Hospital of Navy Medical University, Shanghai, China; ^8^ Department of Surgery, Ohio State University, Wexner Medical Center, Columbus, OH, United States; ^9^ Faculty of Medicine, the Chinese University of Hong Kong, Shatin, Hong Kong, SAR, China

**Keywords:** hepatocellular carcinoma, hepatitis C virus, alpha-fetoprotein, lens culinaris agglutinin-reactive fraction of alpha-fetoprotein, protein induced by vitamin K absence or antagonist-II, diagnosis, biomarker

## Abstract

**Background:**

The GALAD and ASAP scores are two well-recognized algorithms to estimate the risk of hepatocellular carcinoma (HCC) based on gender, age, alpha-fetoprotein (AFP), protein induced by vitamin K absence or Antagonist-II (PIVKA-II) and AFP-L3 (included in the GALAD score but not in the ASAP score). The current study sought to compare the diagnostic performance of each score to detect HCC among patients infected with hepatitis C virus (HCV).

**Methods:**

A multicenter case-control study was undertaken in which blood samples were collected from HCVinfected patients with and without HCC. Using the area under the receiver operating characteristic curve (AUROC), ASAP and GALAD scores were compared relative to diagnostic performance to detect any stage HCV-HCC and early-stage HCV-HCC.

**Results:**

The analytic cohort included 168 HCV-HCC patients and a control group of 193 HCV-infected patients. The ASAP score had a higher AUROC to detect any stage HCV-HCC versus the GALAD score, both in the overall group (0.917 vs. 0.894, P=0.057) and in the cirrhosis subgroup (0.909 vs. 0.889, P=0.132). Similar results were noted relative to the detection of early-stage HCV-HCC, whether defined by BCLC staging (stage 0-A: 0.898 vs. 0.860, *P*=0.026) or 8^th^ TNM staging (stage I: 0.899 vs. 0.870, *P*=0.070). In subgroup analysis to detect AFP-negative HCV-HCC, the ASAP score also demonstrated a higher AUROC than the GALAD score to detect any stage HCV-HCC in the AFP-negative subgroup (0.815 vs. 0.764, *P*=0.063).

**Conclusions:**

The ASAP score had better diagnostic performance for early detection of HCV-HCC compared with the GALAD score. The ASAP score may be preferrable to the GALAD score for HCC screening and surveillance among HCV-infected patients.

## Introduction

Hepatocellular carcinoma (HCC) is the most common primary liver malignancy with most patients developing HCC due to chronic liver diseases. Unfortunately, HCC has a morality-to-incidence ratio that approaches 1 (1). Of note, HCC cases in China accounted for roughly 50% of the new liver cancer cases and deaths that occurred worldwide during 2012 (2). Among the etiological factors associated with HCC, hepatitis C virus (HCV) infection is a common cause. According to the World Health Organization statistics, the global HCV infection rate is about 3%, and there are about 180 million people infected with HCV globally with about 40~60 million cases in China (3–5). Patients with HCV infection have a 2% annual risk and a 7% to 14% five-year risk of developing HCC (6). The benefits of HCC surveillance on survival of HCC at any stage, particularly at early stages, have been codified in the many guidelines recommending HCC surveillance for patients with chronic hepatitis B virus (HBV) and HCV infection (7–9). The early diagnosis of HCC is essential to initiate curative treatments to improve short-term and long-term prognosis. Therefore, highly-effective methods are needed to detect HCC at an earlier stage (10, 11).

Although imaging techniques such as ultrasound and magnetic resonance imaging (MRI) have markedly improved the accuracy of HCC diagnosis, their applications have been limited due to disadvantages such as high cost, invasiveness, and insensitivity to small tumors (12). Serum biomarkers play an essential role in diagnosing HCC, as biomarkers are often more convenient, inexpensive, non-invasive, and reproducible (13–15). Alphafetoprotein (AFP) is a widely used biomarker for HCC diagnosis. The diagnostic accuracy of AFP is limited, however, due to its high false-negative rate to detect small or early-stage tumors. As previous studies have demonstrated, the sensitivity of AFP among patients with HCC was 52% for tumors > 3cm and dropped to only 25% for tumors < 3cm. In addition, AFP may also be elevated in some benign liver diseases, such as chronic hepatitis and cirrhosis even in the absence of HCC (16). Therefore, the application of AFP in the early screening of HCC has been controversial (17). Over the years, novel biomarkers for HCC have been suggested, such as prothrombin induced by vitamin K absence-II (PIVKA-II, also known as des-gamma-carboxy prothrombin) and *lens culinaris* agglutinin-reactive fraction of alpha-fetoprotein (AFP-L3%) (13). These two biomarkers have similar limitations in clinical application due to their insufficient diagnostic performance if used alone (18–20).

Therefore, employing a combined multiple biomarkers approach is essential to improve the accuracy of early HCC diagnosis and reduce the missed diagnosis rate. To this end, Johnson et al. developed a serum-based tool (GALAD) to detect HCC based on objective measures including gender, age, and three serologic biomarkers (i.e., AFP, AFP-L3%, and PIVKA-II) (21). More recently, our team developed the ASAP score comprised of age, sex, AFP, and PIVKA-II based on a Chinese population of HBV-infected patients (22). The reason why AFP-L3 was not enrolled in the ASAP score was that AFP-L3 was not an independent predictor in the regression analysis used to construct the prediction algorithm of the ASAP score (22). Despite having one less predictive variable, the ASAP score performed better than the GALAD score to diagnosis HBV-HCC (AUC: 0.935 vs. 0.921, *P* < 0.006).^22]^ However, the ASAP score has not been verified in any other etiological factors of HCC. In particular, the diagnostic performance of the ASAP score to detect HCC among patients with other types of chronic liver diseases, such as HCV infection, remains unknown. In addition, whether the ASAP score or the GALAD score is more suitable to detect HCC, particularly early-stage HCC among Chinese HCV-infected patients, remains unclear.

Therefore, the current study sought to assess the diagnostic performances of the ASAP score, the GALAD score, and AFP, AFP-L3, and PIVKA-II to detect early HCC in a large cohort of Chinese patients with HCV infection. In addition, we determined and compared the diagnostic performances of the ASAP score versus GALAD score to detect HCC, irrespective of the presence of liver cirrhosis. Specific subgroup analyses were performed to assess detection of early-stage HCC relative to suitable biomarkers or panels of biomarkers that were candidates for HCC screening and surveillance among high-risk populations.

## Methods

### Patients selection and study design

Patients from March 2018 to May 2021 with chronic HCV infection irrespective of the presence of HCC were retrospectively identified from the databases of four Chinese hospitals (Zhejiang University Lishui Hospital, Eastern Hepatobiliary Surgery Hospital of Shanghai, the First Affiliated Hospital of Soochow University, and Zhejiang Provincial People’s Hospital). Patients who met any of the following exclusion criteria were excluded from the study: 1) less than 18 years old, 2) classified as Barcelona Clinic Liver Cancer (BCLC) stage D, 3) unknown etiology of HCC, 4) HBV and hepatitis C virus (HCV) co-infection, 5) receipt of any anti-HCC treatment before blood collection, 6) incomplete medical record or missing data on variables and outcomes including HCC biomarkers test. HCV infection was defined as HCV-RNA positivity within six months prior to surgery. HCC was diagnosed with imaging including ultrasound, computerized tomography, and magnetic resonance imaging and confirmed by biopsy or postoperative histopathological examination (9). Cirrhosis was diagnosed based on clinical evidence of portal hypertension or hepatic decompensation according to the established guidelines (9). HCV-HCC was defined as HCC among patients with HCV infection but without evidence of any other underlying liver diseases. Two tumor staging systems were used to determine the stage of disease: BCLC staging and 8^th^ edition American Joint Committee on Cancer tumor-node-metastasis (TNM) staging. Early-stage HCC was defined as BCLC stage 0+A and/or 8^th^ edition TNM stage I. Written informed consent was obtained from all participants of this study. The ethics committee approved the study of each study site, and the study conduction conformed to the ethical guidelines of the 1975 Declaration of Helsinki.

### Measurements of tumor biomarkers

Peripheral blood samples were collected from each participant before any HCC treatment. Serum samples were separated from the blood samples by centrifugation at 700g for 10 min. Serum samples were subsequently aliquoted and frozen at -80°C until testing. The sample storage facilities and conditions were standardized at each study site. Serum samples were sent to the Abbott testing centers on a regular basis, and liquid nitrogen was used during transportation. AFP and PIVKA-II serum concentrations were measured with the commercially available ARCHITECT immunoassay per the defined protocol (Abbott Diagnostics). Serum measurements of AFP-L3% were determined utilizing the Fujirebio assay (Fujirebio Diagnostics). The lower limits of detection for AFP, PIVKA-II, and AFP-L3% assays were 0.6 ng/mL, 5.0 mAU/mL, and 0.5%, respectively. The technicians performing the laboratory tests were blinded to the diagnosis of the participants. No adverse events related to serum sample collection were observed.

### Statistical Analysis

The ASAP score was calculated using the following equation: ASAP score = -7.58 + 0.05 × age - 0.58 × gender + 0.42 × Ln (AFP [ng/ml]) + 1.11 × Ln (PIVIKA-II [mAU/ml]), where gender = 0 for males and 1 for females. The GALAD score was calculated using the following equation: GALAD score = -10.08 + 0.09 × age + 1.67 × gender + 2.34 × Lg (AFP [ng/ml]) + 0.04 × AFP-L3%% + 1.33 × Lg (PIVKA-II [mAU/ml]), where gender = 0 for females and 1 for males.

Data count distributions were compared between groups using the χ^2^ test, with Fisher’s exact test utilized for small sample sizes. To ensure that the normality assumption was met, measurement data were compared between groups using the student’s *t*-test and the analysis of the variance model on the log scale. Categorical variables were expressed as percentages and continuous variables as means (standard deviations) or medians (interquartile ranges). The receiver operating characteristic (ROC) curves were used to determine the area under the curve (AUC) for AFP, PIVKA-II, or AFP-L3% alone and for combinations of two or three biomarkers to predict HCC. Comparisons among AUC values were performed using DeLong’s test (23). To evaluate the diagnostic performances of biomarker combinations, binary logistic regression was used to predict the probability of developing HCC. The AUC, sensitivity, specificity, positive predictive value (PPV), and negative predictive value (NPV) were used to report diagnostic performances. All statistical analysis were performed using Medcalc version 19.6.3 (MedCalc Software, Ostend, Belgium) for Windows and SPSS software version 25.0 (SPSS Inc., an IBM Company, Chicago, IL, USA). A two-tailed value of *P* < 0.05 was considered statistically significant.

## Results

### Clinical variables of enrolled patients

**Figure 1:**
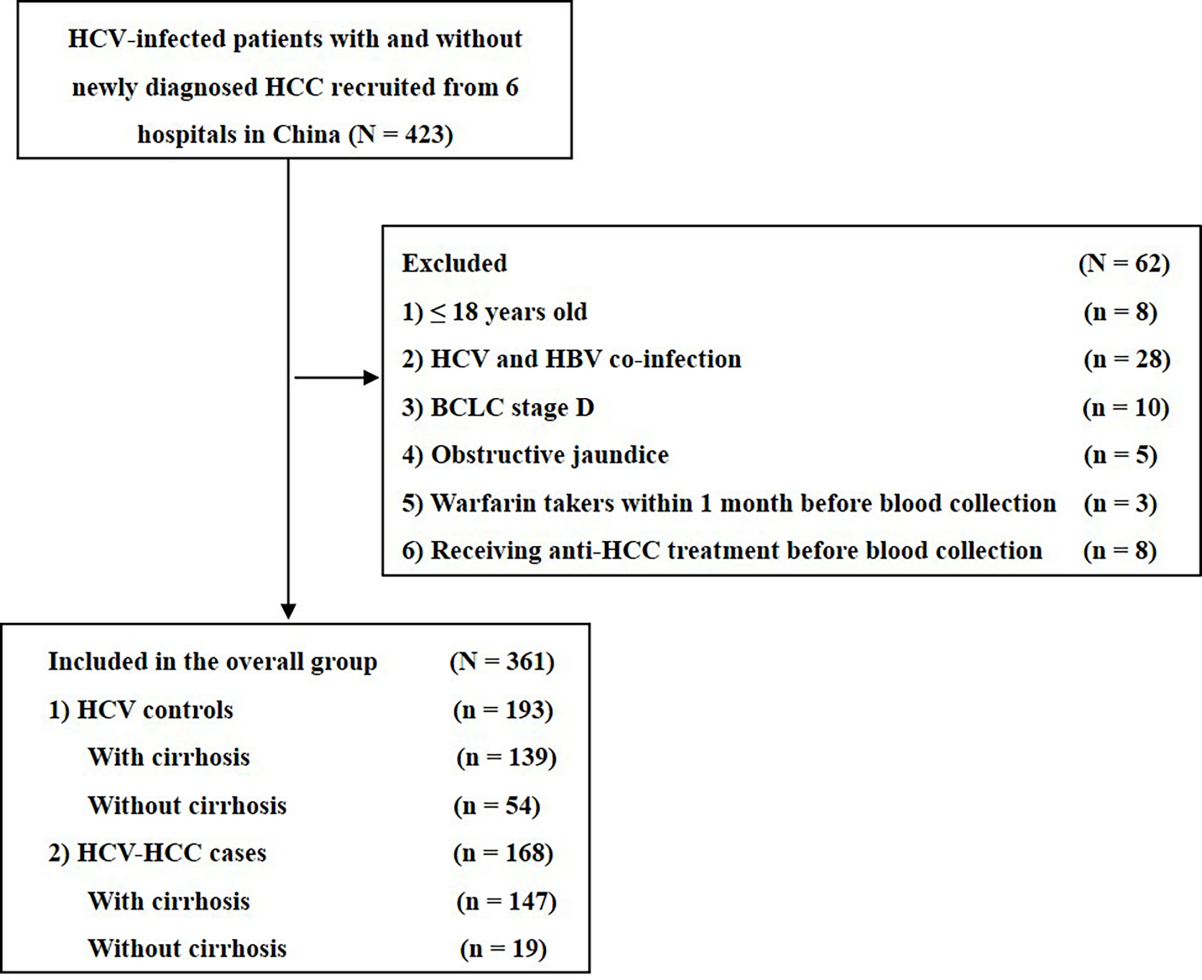
Flow chart of the study.

**Figure 2:**
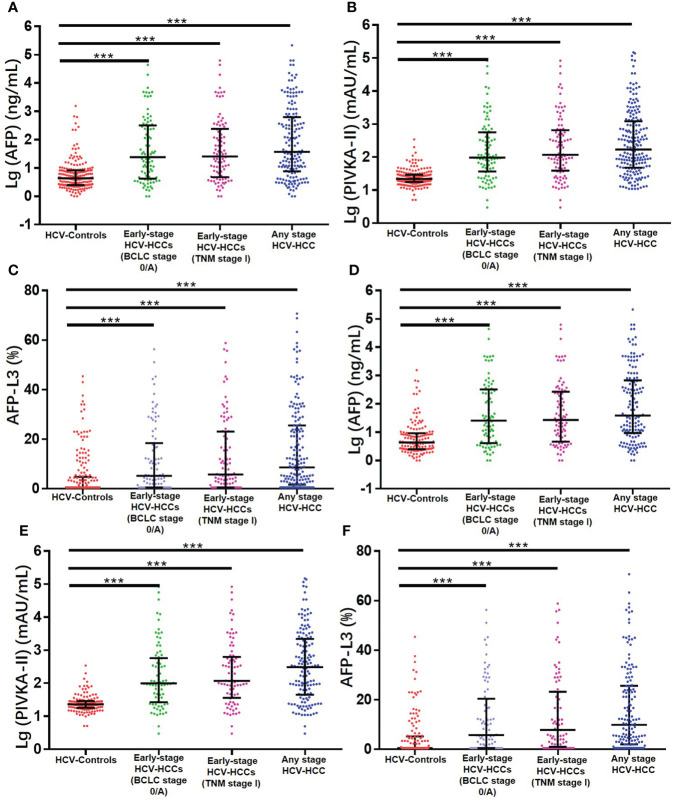
Serum concentrations of three serum biomarkers in the overall group **(A)** AFP; **(B)** PIVKA-II; **(C)** AFP-L3% and in the cirrhosis subgroup **(D)** AFP; **(E)** PIVKA-II; **(F)** AFP-L3%. ***P < 0.001.

Among 585 HCV-infected patients, 361 patients met eligibility criteria and were enrolled into the study (**Figure 1**). The clinical characteristics of the HCV-HCC and HCV-control groups are summarized in **Table 1**. Several clinical characteristics differed among patients in the HCV-HCC versus HCV groups including age, sex, Child-Pugh grading, cirrhosis, individual biomarkers of AFP, PIVKA-II, and AFP-L3%, and diagnostic scores of ASAP and GALAD, with all *P* < 0.05. Of note, median levels of the tumor biomarkers AFP, PIVKA-II, and AFPL3% were higher among HCV-HCC patients versus HCV-control patients (37.1 versus 4.3 ng/mL for AFP, 288.0 versus 22.0 mAU/mL for PIVKA-II, and 8.6% versus 0.5% for AFP-L3%; all *P* < 0.05) (**Table 1**, and **Figure 2**).

**Table 1:** Clinical characteristics of the overall group.

N (%)	HCV-HCC (N = 168)	HCV controls (N = 193)	*P* value
Baseline characteristics			
Age, years*	62.0 ± 8.5	56.2 ± 12.2	< 0.001
Male sex	123 (73.2)	115 (59.6)	< 0.001
Cirrhosis	147 (87.5)	139 (72.0)	< 0.001
Child-Pugh grade			
A	154 (91.7)	177 (91.7)	< 0.001
B/C	14 (11.4)	16 (7.4)	
Platelet, ×10^9^/L*	162 ±92.9	148.6±86.2	0.450
Bilirubin, μmol/L*	14.5 (11.3, 24.8)	15.3 (11.9, 21.9)	0.781
Albumin, g/L*	44.0 ± 5.6	45.2	0.128
AFP, ng/mL*	37.1	4.3 (2.5, 8.2)	0.0052
Negative (< 20 ng/mL)	68 (40.5)	173 (89.6)	< 0.001
Positive (≥20 ng/mL)	100 (59.5)	20 (16.3)	
PIVKA-II, mAU/ml*	288.0 (47.1, 932.0)	22.0 (19.0, 28.9)	0.005
Negative (< 40 mAU/mL)	39 (23.2)	168 (87.1)	< 0.001
Positive (≥ 40 mAU/mL)	129 (76.8)	25 (12.9)	
AFP-L3, %*	8.6 (0.5, 12.5)	0.5 (0.5, 5.4)	< 0.001
Negative (< 10%)	87 (51.8)	155 (80.3)	< 0.001
Positive (≥ 10%)	81 (48.2)	38 (19.7)	
ASAP score	3.10 (0.57, 5.23)	-1.05 (-1.49, 0.35)	< 0.001
GALAD score	4.50 (2.05, 5.87)	-0.35 (-0.51, 0.53)	< 0.001
Tumor characteristics			
Largest tumor size, cm*	5.6 ± 3.6		
> 3.0 cm	118 (70.2)		
Multiple tumors	148 (88.1)		
Macrovascular invasion	28 (16.7)		
Extrahepatic metastasis	17 (10.1)		
BCLC stage			
0/A (Early stage)	91 (54.2)		
B/C (Intermediate/advanced stage)	75 (45.8)		
TNM stage (the 8^th^ Edition)			
I (Early stage)	92 (54.8)		
II+III (Intermediate/advanced stage)	74 (45.2)		

*Values are mean ± standard deviation or median with interquartile range.

AFP, alpha-fetoprotein; AFP-L3, lens culinaris agglutinin A-reactive fraction of alpha-fetoprotein; BCLC, Barcelona Clinic Liver Cancer; HCC, hepatocellular carcinoma; HCV, hepatitis C virus; PIVKA II, protein induced by vitamin K absence or antagonist-II; TNM, tumor mode metastasis.

### Diagnostic performances for any stage HCV-HCC

**Figure 3:**
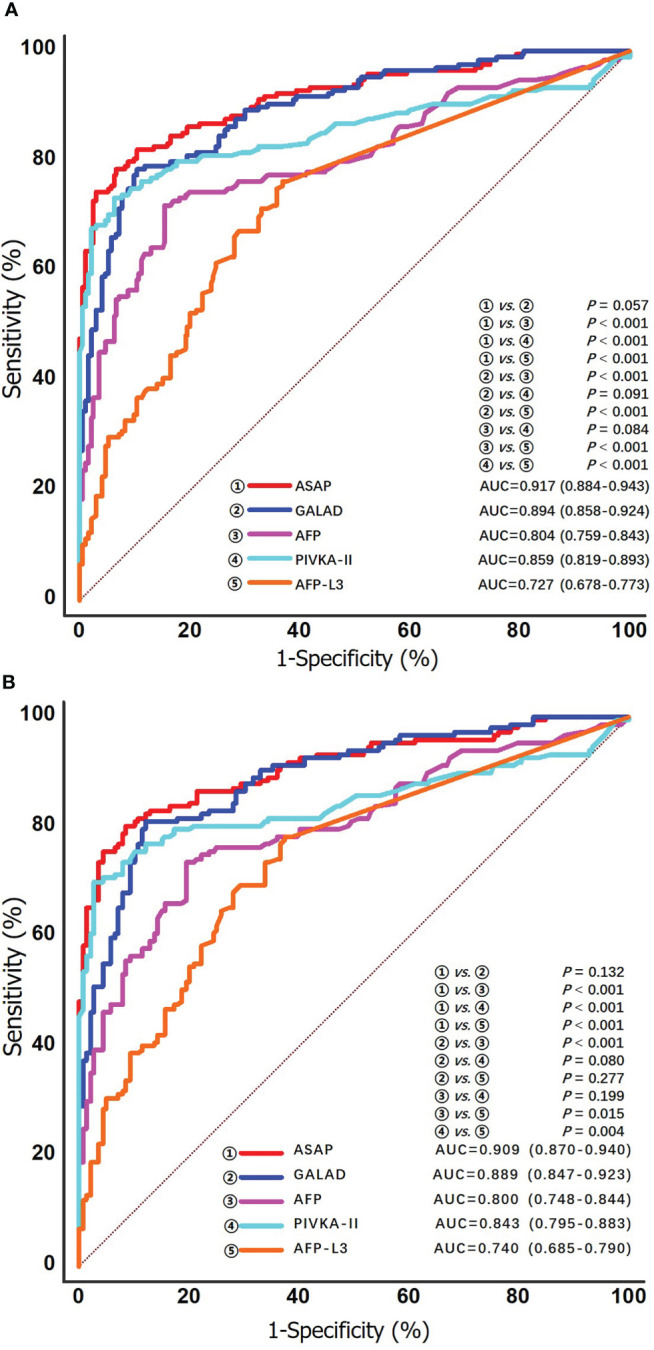
ROC curves of the ASAP score, the GALAD score, AFP, PIVKA-II and AFP-L3% for detecting any stage HCV-HCC in the overall group **(A)** and the cirrhosis subgroup **(B)**.

ROC curve analysis was used to evaluate the performance of individual biomarkers, as well as combination of the two prediction models. As shown in **Table 2** and **Figure 3**, both ASAP and GALAD demonstrated better diagnostic performance to diagnose HCC than the three individual biomarkers alone. The sensitivity, specificity, PPV, and NPV were presented in **Table 2**. Using 20 ng/mL for AFP, 40 mAU/mL for PIVKA-II, and 10% for AFP-L3% as the clinical cut-off values, the sensitivity of AFP, PIVKA-II, and AFP-L3% to detect HCC among patients with HCV-HCC were 59.5%, 76.8%, and 48.2%, respectively; the specificity was 89.6%, 87.1%, and 80.3%, respectively. Of note, the AUC of PIVKA-II alone to -HCC was 0.859 (0.819-0.893), which was better than the performance of AFP alone (0.804 [0.759-0.843]; *P* = 0.084) or AFP-L3% alone (0.727 [0.678-0.773], *P* <0.001. The ASAP score had a similar diagnostic ability compared with the GALAD score (AUC of ASAP = 0.917 [0.884-0.943]; AUC of GALAD = 0.894 [0.858-0.924]).

**Table 2:** Diagnostic performances of the ASAP score, the GALAD score, AFP, PIVKA-II, and AFP-L3% for detecting any stage HCV-HCC in the overall group and the cirrhosis subgroup.

		AUC (95 %CI)	*P* value (ASAP vs. Others)	Sensitivity (%)(95 % CI)	Specificity (%)(95 % CI)	PPV (%) (95 % CI)	NPV (%) (95 % CI)	Positive LR	Negative LR
**Overall Group: Any stage HCV-HCC cases (n = 168) vs HCV controls (n = 193)**									
**ASAP score**		0.917 (0.884-0.943)	Reference	78.6 (71.6-84.5)	93.3 (88.8-96.4)	91.0 (85.7-94.5)	83.3 (78.9-87.0)	11.7	0.2
**GALAD score**		0.894 (0.858-0.924)	0.057	78.6 (71.6-84.5)	89.6 (84.4-93.6)	86.8 (81.2-91.0)	82.8 (78.2-86.6)	7.6	0.2
**AFP**		0.804 (0.759-0.843)	<0.001	59.5(51.7-67.)	89.6 (84.4-93.6)	83.3 (76.4-88.5)	71.8 (67.8-75.5)	5.7	0.5
**PIVKA-II**		0.859 (0.819-0.893)	<0.001	76.8 (69.7-82.9)	87.1 (81.5-91.4)	83.8 (78.0-88.2)	81.2 (76.5-85.1)	5.9	0.3
**AFP-L3%**		0.727 (0.678-0.773)	<0.001	48.2 (40.5-56.0)	80.3 (74.6-86.1)	68.6 (61.2-75.3)	64.2 (60.4-67.8)	2.5	0.6
**Cirrhosis Subgroup: Any stage HCV-HCC cases (n = 147) vs. HCV controls (n = 139)**									
**ASAP score**	0.909 (0.857-0.931)		Refrence	80.3 (69.6-87.1)	91.4 (85.4-95.5)	90.8 (85.1-94.4)	81.4 (75.9-85.9)	9.3	0.2
**GALAD score**	0.889 (0.847-0.923)		0.132	81.0 (73.7-87.0)	87.8 (81.1-92.7)	87.5 (81.7-91.7)	81.3 (75.6-85.9)	6.6	0.2
**AFP**	0.800 (0.748-0.844)		<0.001	60.5 (52.2-68.5)	86.3 (79.5-91.6)	82.4 (75.1-87.9)	67.4 (62.6-71.9)	4.4	0.5
**PIVKA-II**	0.843 (0.795-0.883)		<0.001	76.9 (69.2-83.4)	84.9 (77.8-90.4)	84.3 (78.2-89.0)	77.6 (71.9-82.5)	5.1	0.3
**AFP-L3 %**	0.740 (0.685-0.790)		<0.001	50.3 (42.0-58.7)	81.3 (73.8-87.4)	74.0 (66.0-80.7)	60.8 (56.4-65.0)	2.7	0.6

AFP, alpha-fetoprotein; AFP-L3, lens culinaris agglutinin A-reactive fraction of alpha-fetoprotein; AUC, area under the curve; CI, confidence interval; HCC, hepatocellular carcinoma; HCV, hepatitis C virus; LR, likelihood ratio; NPV, negative predictive value; PIVKA-II, protein induced by vitamin K absence or antagonist-II; PPV, positive predictive value.

### Diagnostic performances for any stage HCV-HCC among the subgroup of patients with cirrhosis

HCV patients without HCC had a markedly lower prevalence of cirrhosis compared with HCV-HCC patients (72.0% vs. 87.5%, *P* < 0.05). Further analyses to assess the diagnostic performance of the ASAP score, the GALAD score, AFP, PIVKA-II, and AFP-L3% to detect HCV-HCC among patients with HCV-cirrhosis (HCC, n = 147; control group, n = 139) were then performed. ROC curve analysis was then used to assess the performance of the ASAP score, the GALAD score, and AFP, PIVKA-II, or AFP-L3% alone to diagnose HCV-HCC among patients with HCV-cirrhosis (**Figure 3**). Similar to the overall cohort, subgroup analysis of patients with HCVcirrhosis demonstrated that the ASAP score and the GALAD score had higher sensitivity (80.3% and 81.0%, respectively) than any individual biomarker (60.5% for AFP, 76.9% for PIVKA-II, and 50.3% for AFP-L3%, respectively). The ASAP score had a better diagnostic ability versus the GALAD score with a higher AUC of 0.909 (0.870-0.940) (*P* < 0.001) among patients with HCC-cirrhosis (**Table 2**).

### Diagnostic performances for early-stage HCV-HCC

**Figure 4:**
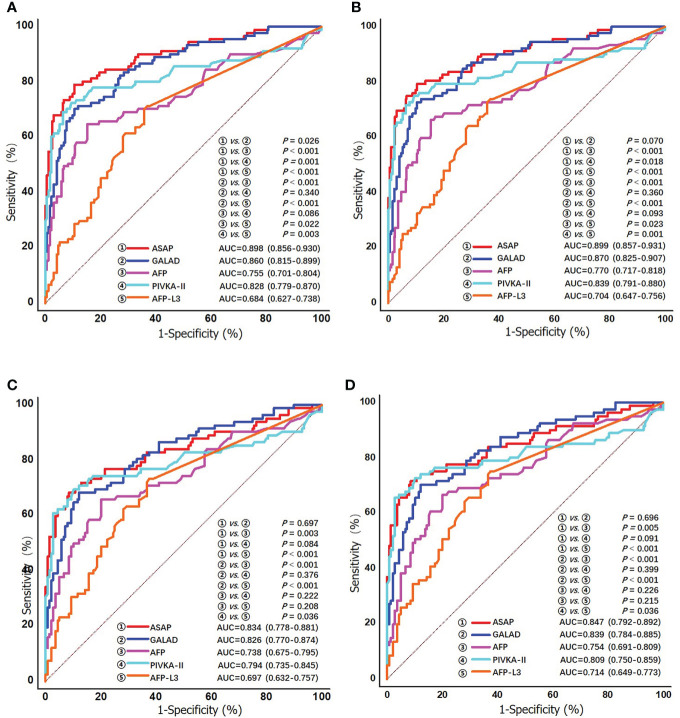
ROC curves of the ASAP score, the GALAD score, AFP, PIVKA-II, and AFP-L3% for detecting early stage HCV-HCC in the overall group **(A)** BCLC stage 0/A; and **(B)** TNM stage I; and in the cirrhosis subgroup **(C)** BCLC stage 0/A; and **(D)** TNM stage I.

Clinical characteristics of early-stage HCV-HCC according to the BCLC and TMN staging systems are shown in **Supplement Table 1**. The diagnostic performance of the ASAP score, the GALAD score, AFP, PIVKA-II, and AFP-L3% to detect early-stage HCV-HCC was evaluated and compared. Among patients with early-stage HCC (BCLC stage 0+A, n = 91), PIVKA-II demonstrated better diagnostic performance (AUC 0.828, 0.779-0.870) compared with AFP (AUC 0.755, 0.701-0.804) alone or AFP-L3% (AUC 0.684, 0.627-0.738) alone. In addition, the ASAP score (AUC 0.898, 0.856-0.930) and the GALAD score (AUC 0.860, 0.815-0.899) performed better than any individual biomarker (**Figure 4**). Similar findings were obtained using the 8th TNM staging system to define early-stage HCC. Specifically, the ASAP score and the GALAD score achieved higher AUC of 0.899 (0.857-0.931) and 0.870 (0.825-0.907), respectively, compared with individual biomarkers (AUC ranging from 0.704 to 0.839). Similar results were noted in the subgroups of liver cirrhosis patients. In particular, individual biomarkers and the two models achieved similar results in early-stage subgroups with AUC values ranging from 0.697 to 0.834 when defined by BCLC stage 0+A, and 0.714 to 0.847 when defined by the 8th TNM stage I (**Table 3**, and **Figure 4**). Results of sensitivity, specificity, PPV, NPV, and percent correctly classified when using different cutoff points are presented in **Table 3**.

**Table 3:** Diagnostic performances of the ASAP score, the GALAD score, AFP, PIVKA-II, and AFP-L3% for detecting early-stage HCV-HCC in the overall group and the cirrhosis subgroup.

		AUC (95 %CI)	*P* value (ASAP vs. Others)	Sensitivity (%)(95 % CI)	Specificity (%)(95 % CI)	PPV (%) (95 % CI)	NPV (%) (95 % CI)	Positive LR	Negative LR
**Overall Group: Early-stage HCV-HCCs (BCLC stage 0/A) (n = 91) vs. EHCV controls (n = 193)**									
**ASAP score**		0.898 (0.856-0.930)	Reference	79.1 (69.3-86.9)	89.6 (84.4-93.6)	78.3 (70.1-84.7)	90.1 (85.9-93.2)	7.6	0.2
**GALAD score**		0.860 (0.815-0.899)	0.026	70.3 (59.8-79.5)	89.6 (84.4-93.6)	76.2 (67.4-83.2)	86.5 (82.3-89.8)	6.8	0.3
**AFP**		0.755 (0.701-0.804)	<0.001	53.9 (43.1-64.4)	89.6 (84.4-93.6)	71.0 (60.8-79.5)	80.5 (76.6-83.8)	5.2	0.5
**PIVKA-II**		0.828 (0.779-0.870)	0.001	73.6 (63.3-82.3)	86.0 (80.3-90.6)	71.3 (63.1-78.2)	87.4 (83.0-90.7)	5.3	0.3
**AFP-L3%**		0.684 (0.627-0.738)	<0.001	41.8 (31.5-52.6)	80.8 (74.6-86.1)	50.7 (41.3-60.0)	74.6 (70.9-78.0)	2.2	0.7
**Overall Group: Early-stage HCV-HCCs (TNM stage I) (n = 92) vs. HCV controls (n = 193)**									
**ASAP score**	0.899 (0.857-0.931)		Reference	79.4 (69.6-87.1)	89.6 (84.4-93.6)	78.5 (70.4-84.8)	90.1 (85.9-93.2)	7.7	0.2
**GALAD score**	0.870 (0.825-0.907)		0.070	72.8 (62.6-81.6)	89.6 (84.4-93.6)	77.0 (68.5-83.8)	87.4 (83.2-90.7)	7.0	0.3
**AFP**	0.770 (0.717-0.818)		<0.001	55.4 (44.7-65.8)	89.1 (83.8-93.1)	70.8 (60.9-79.1)	80.8 (76.9-84.1)	5.1	0.5
**PIVKA-II**	0.839 (0.791-0.880)		0.018	76.1 (66.1-84.4)	85.5 (79.7-90.1)	71.4 (63.5-78.2)	88.2 (83.8-91.6)	5.2	0.3
**AFP-L3 %**	0.704 (0.647-0.756		<0.001	44.6 (34.2-55.3)	80.8 (74.6-86.1)	52.6 (43.4-61.6)	75.4 (71.6-78.8)	2.3	0.7
**Cirrhosis Subgroup: Early-stage HCV-HCCs (BCLC stage 0/A) (n=82) vs. HCV-cirrhosis controls (n=139)**									
**ASAP score**	0.834 (0778-881)		Reference	68.3 (57.1-78.1)	91.4 (85.4-95.5)	82.4 (72.7-89.1)	83.0 (78.0-87.1)	7.9	0.4
**GALAD score**	0.826 (0770-0.874)		0.697	68.3 (57.1-78.1)	87.8 (81.1-92.7)	76.7 (67.3-84.0)	82.4 (77.2-86.6)	5.6	0.4
**AFP**	0.738 (0.675-0.795)		0.003	65.9 (54.6-76.0)	79.9 (72.2-86.0)	65.9 (57.2-73.5)	79.9 (74.4-84.4)	3.3	0.4
**PIVKA-II**	0.794 (0.735-0.845)		0.084	69.5 (58.4-79.2)	89.9 (83.7-94.4)	80.3 (70.8-87.2)	83.3 (78.2-87.4)	6.9	0.3
**AFP-L3%**	0.697 (0.632-0.757)		< 0.001	73.2 (62.2 - 82.4)	62.6 (54.0 - 70.6)	53.6 (47.3 - 59.7)	79.8 (73.0-85.3)	2.0	0.4
**Cirrhosis Subgroup: Early-stage HCV-HCCs (TNM stage I) (n = 81) vs. HCV-cirrhosis controls (n = 139)**									
**ASAP score**	0.847 (0.792-0.892)		Reference	71.6 (60.5-81.1)	91.4 (85.4-95.5)	82.9 (73.5-89.4)	84.7 (79.6 - 88.7)	8.3	0.3
**GALAD score**	0.839 (0.784-0.885)		0.696	70.4 (59.2-80.0)	87.8 (81.1-92.7)	77.0 (67.8-84.3)	83.6 (78.3-87.7)	5.8	0.3
**AFP**	0.754 (0.691-0.809)		0.005	66.7 (55.3-76.8)	79.9 (72.2-86.2)	65.9 (57.2-73.5)	80.4 (74.9-85.0)	3.3	0.4
**PIVKA-II**	0.809 (0.750-0.859)		0.091	72.8 (61.8-82.1)	89.9 (83.7-94.4)	80.8 (71.6-87.6)	85.0 (79.8-89.1)	7.2	0.3
**AFP-L3%**	0.714 (0.649-0.773)		< 0.001	75.3 (64.5-84.2)	62.6 (54.0 - 70.6)	54.0 (47.8 - 60.1)	81.3 (74.4-86.7)	2.0	0.4

AFP, alpha-fetoprotein; AFP-L3, lens culinaris agglutinin A-reactive fraction of alpha-fetoprotein; AUC, area under the curve; BCLC, Barcelona Clinic Liver Cancer; CI, confidence interval; HCC, hepatocellular carcinoma; HCV, hepatitis C virus; LR, likelihood ratio; NPV, negative predictive value; PIVKA-II, protein induced by vitamin K absence or antagonist-II; PPV, positive predictive value; TNM, tumor-node-metastasis.

### Diagnostic performances of the ASAP score and the GALAD score for AFP-negative HCV-HCC

**Figure 5:**
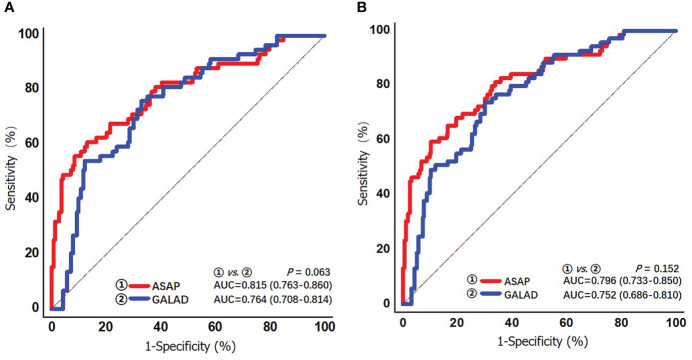
ROC curves of the ASAP score and the GALAD score for detecting AFP-negative HCV-HCC (AFP < 20 ng/mL) in the overall group **(A)** and the cirrhosis subgroup **(B)**.

The diagnostic performances of ASAP and GALAD were further examined among HCV-HCC patients who had the HCC diagnosis missed using AFP alone. In particular, the diagnostic performances of the ASAP and GALAD scores in subgroups of HCV-HCC and HCC-cirrhosis among AFP-negative patients was evaluated. As noted in **Table 4** and **Figure 5**, the ASAP score demonstrated a better ability to distinguish AFP-negative HCC from HCV controls than the GALAD score (AUC of ASAP = 0.815 vs. AUC of GALAD = 0.764, *P* < 0.01) in the subgroups of patients with HCV-HCC; in fact, the sensitivity was 59.4% versus 73.9% and the specificity was 89.6% versus 70.0%. Moreover, the ASAP score demonstrated a better performance to discriminate AFP-negative HCC from HCC arising in the setting of cirrhosis (AUC of ASAP = 0.796 vs. AUC of GALAD = 0.752, *P* < 0.01) compared with the GALAD score with a sensitivity of 61.2% versus 76.3% and specificity of 87.1% versus 66.9%, respectively.

**Table 4:** Diagnostic performances of the ASAP and the GALAD score for detecting AFP-negative HCV-HCC (AFP<20 ng/ml) in the overall group and the cirrhosis subgroup.

	AUC (95 % CI)	*P* value	Sensitivity (%) (95 % CI)	Specificity (%) (95 % CI)	PPV (%) (95 % CI)	NPV (%) (95 % CI)	Positive LR	Negative LR
**Overall Subgroup: AFP-negative HCV-HCCs (n = 69) vs. HCV controls (n = 193)**								
**ASAP score**	0.815 (0.763-0.860)	Reference	59.4 (46.9-71.1)	89.6 (84.4-93.6)	67.2 (56.4-76.4)	86.1 (82.2-89.2)	5.7	0.5
**GALAD score**	0.764 (0.708-0.814)	0.063	73.9 (61.9-83.7)	70.0 (62.9-76.3)	46.8 (40.5-53.2)	88.2 (83.3-91.9)	2.5	0.4
**Cirrhosis Subgroup: AFP-negative HCCs (n = 59) vs. HCV-cirrhosis controls (n = 139)**								
**ASAP score**	0.796 (0.733-0.850)	Reference	61.02 (47.4-73.5)	87.1 (80.3-92.1)	66.7 (55.4-76.3)	84.0 (79.2-87.9)	4.7	0.5
**GALAD score**	0.752 (0.686-0.810)	0.512	76.3 (63.4-86.4)	66.9 (58.4-74.6)	49.5 (42.6-56.3)	86.9 (80.6-91.4)	2.3	0.4

AFP, alpha-fetoprotein; AFP-L3, lens culinaris agglutinin A-reactive fraction of alpha-fetoprotein; AUC, area under the curve; BCLC, Barcelona Clinic Liver Cancer; CI, confidence interval; HCC, hepatocellular carcinoma; HCV, hepatitis C virus; LR, likelihood ratio; NPV, negative predictive value; PIVKA-II, protein induced by vitamin K absence or antagonist-II; PPV, positive predictive value.

## Discussion

HCC surveillance is recommended especially among high-risk individuals as the mortality-to-incidence ratio of this disease is approaching one and curative-intent therapeutic options are limited among patients who have already progressed to intermediate or advanced stage disease when HCC is detected (9, 24). In the current multicenter study, the ASAP score demonstrated comparable diagnostic ability to the GALAD score among HCVinfected patients for HCC detection; in addition, the ASAP score had better ability than any individual biomarkers including AFP, PIVKA-II and AFP‐L3%. Similar results were noted in subgroup analyses among patients with HCV-cirrhosis, as well as patients with early-stage HCV-HCC, irrespective of which staging system (BCLC or TNM) was adopted. From the public or global health standpoint, both the ASAP score and the GALAD score can be used in parts of the world where medical resources are limited, and liver ultrasound is not widely available or easily affordable. One main advantage of these scores is that each is easy to calculate and can serve as excellent screening tests. As such, utilization of these diagnostic scores may increase the uptake of and compliance with surveillance and consequently improve the effectiveness of surveillance programs among at-risk populations of HCC.

To achieve early detection of HCC, serological AFP and liver ultrasound are conventionally recommended (25, 26). However, ultrasound interpretation is operator-dependent and can be problematic in patients with central obesity or underlying cirrhosis (27). A recent single-center study analyzing 941 patients with cirrhosis reported that ultrasound alone was inadequate to exclude HCC in up to 20% of patients (28). As such, serologic biomarkers are needed to complement ultrasound to detect HCC (29). Use of serologic biomarkers scores may decrease the risk of surveillance-related variation associated with false positive ultrasound results, while also maximizing the potential benefits of early HCC detection by identifying patients with false negative ultrasound results (28). Serum biomarkers are promising tools for surveillance and early diagnosis of HCC owing to their noninvasive, objective, and reproducible characteristics. Among many proposed biomarkers, AFP, PIVKA-II, and AFP‐L3% are the HCC-specific biomarkers commonly used in current clinical practice. Although AFP has been widely used as a serum biomarker of tumor response for HCC, one of its significant limitations is that approximately 30-50% of patients with HCC are AFP “non-secretors” (30, 31). In fact, we noted that 43.7% of patients in our cohort were AFP non-secretors, and in this subgroup, PIVKA-II was a useful alternative serum biomarker as changes in its levels tracked with treatment response in 65% of AFP non-secretors. Data from the current study suggested that as an individual biomarker, PIVKA-II demonstrated the ability to diagnose HCV-HCC accurately; moreover, the diagnostic ability of PIVKA-II was better than that of AFP or AFP‐L3% used alone, with higher AUC (0.859 [0.819-0.893]) values and greater sensitivity (76.8% [69.7-82.9%]) and specificity (87.1% [81.5-91.4%]) than the other two biomarkers. PIVKA-II had comparable diagnostic efficacy for HCC detection independent of AFP-positive or negative status, making PIVKA-II a valuable supplement to AFP assessments. These conclusions were consistent with several other studies (32–34). Given that individual biomarkers are likely insufficient to detect HCC, utilizing combinations of these complementary markers may be helpful to diagnose HCC.

Johnson P. J. et al. developed a serum-based tool (i.e., the GALAD model and associated GALAD score) for surveillance of HCC based on a UK cohort (21, 35). In 2019, the ASAP score was first developed based on participants recruited from 11 Chinese hospitals using a statistical model that could determine the risk of developing HCC in individual HBV patients using objective measures, which were mainly serological tumor markers (22). The ASAP score included age, sex, AFP, and PIVKA-II, while the GALAD score utilized age, sex, AFP, PIVKA-II, and AFP-L3%. AFP-L3 is one of the three glycosylated forms of AFP, and the level of AFP-L3% largely depends on the level of AFP. However, at the most frequently used cut-off value (10%), AFP-L3% has a specificity of 99.4% with a low sensitivity of 18.8%, indicating a poor ability to diagnose HCC as sensitivity takes priority over specificity in surveillance (36, 37). For the diagnosis of early-stage HCC, AFP-L3% is not recommended because of the need for an elevated AFP level, which limits its effectiveness; while AFP-L3% may serve as a supplementary for AFP, highly sensitive AFP-L3% measurements are region-restricted and costly (38, 39). As such, we constructed the ASAP score excluding AFP-L3% because the measurement of this marker is complex, time-consuming, expensive, and requires up to a 400-μL volume of serum sample; in addition, the contribution of AFP-L3% to risk prediction of HCC was low. As demonstrated in the current study’s results, the ASAP score performed comparably to the GALAD score. Furthermore, the ASAP score had a better discriminatory ability than the GALAD score in subgroup analyses of patients with HCV-cirrhosis and patients with early-stage HCV-HCC.

From the public or global health standpoint, both the ASAP score and the GALAD score can be used in parts of the world where medical resources are limited, and liver ultrasound is not widely available or easily affordable. One main advantage of these scores is that each is easy to calculate and can serve as excellent screening tests. As such, utilization of these diagnostic scores may increase the uptake of and compliance with surveillance and consequently improve the effectiveness of surveillance programs among at-risk populations of HCC.

The current study had several limitations. The retrospective study design had the inherent defect of selection bias. The present study population’s viral status (in particular, the serum HCV-RNA levels) was unknown. The study failed to account for anti-HCV therapy treatment history/status could have impacted the results. Furthermore, the diagnostic performances between liver ultrasound and serum biomarkers could not be compared in the present study. Considering that the GALAD score was constructed based on an UK cohort and the ASAP score was built on the basis of a Chinese cohort, the AUC of the ASAP score may have had a slight advantage with a database of Chinese patients used for the model validation. These two prediction models have not been compared in the international setting or relative to different stages of disease. In addition, the diagnostic performance of these two scores among HCC patients with other etiologies of chronic liver diseases, such as HBV infection, alcoholic liver disease, or nonalcoholic fatty liver disease, deserves to further evaluate in the future.

## Conclusion

The ASAP score demonstrated excellent diagnostic performance among a large cohort of Chinese patients with HCV-HCC. The ASAP score had comparable or even better diagnostic ability compared with the GALAD score to detect any stage and early-stage HCV-HCC. In the future, comparison of the ASAP score with the GALAD score will be performed in a more extensive prospective multicenter cohort study and the costeffectiveness of the ASAP score versus the GALAD score with/without AFP-L3% will be further investigated before the comprehensive implementation of the prediction model in clinical practice.

## Data availability statement

The raw data supporting the conclusions of this article will be made available by the authors upon request, without undue reservation.

## Ethics statement

The studies involving human participants were reviewed and approved by The ethic committee of the Lishui Municipal Central Hospital. Written informed consent for participation was not required for this study in accordance with the national legislation and the institutional requirements. Written informed consent was not obtained from the individual(s) for the publication of any potentially identifiable images or data included in this article.

## Author contributions

S-YL, CL, L-YS, and M-CG contributed equally to this work. Study concept and design: S-YL, CL, L-YS, M-CG, TP, X-MT, and TY. Drafting the manuscript: S-YL, CL, L-YS, and M-CG. Acquisition of data: S-YL, CL, L-YS, M-CG, LL, HX, M-DW, L-QY, FS, X-MT, and TY. Statistical analysis: S-YL, L-YS, TP, HX, and TY. Approval of the final version manuscript: All authors. Accountable for all aspects of the work: All authors. X-MT and TY had full access to all the data in the study and take responsibility for the integrity of the data and the accuracy of the data analysis. All authors contributed to the article and approved the submitted version.

## Funding

This work was supported in part by the Health Science and Technology Plan of Zhejiang Province (No. 2022KY453 for S-YL), the National Natural Science Foundation of China (No. 81972726 for TY), and Zhejiang Province Administration Foundation of Traditional Chinese Medicine (No. 2020ZB305 for S-YL).

## Conflict of interest

The authors declare that the research was conducted in the absence of any commercial or financial relationships that could be construed as a potential conflict of interest.

## Publisher’s note

All claims expressed in this article are solely those of the authors and do not necessarily represent those of their affiliated organizations, or those of the publisher, the editors and the reviewers. Any product that may be evaluated in this article, or claim that may be made by its manufacturer, is not guaranteed or endorsed by the publisher.

## Abbreviations

HCC, hepatocellular carcinoma; HCV, hepatitis C virus; AFP, alpha-fetoprotein; PIVKAII, protein induced by vitamin K absence or Antagonist-II; AFP-L3%, lens culinaris agglutinin-reactive fraction of alpha-fetoprotein; HBV, hepatitis B virus; BCLC, Barcelona Clinic Liver Cancer; TNM, tumor-node-metastasis; ROC, receiver operating characteristic; AUC, area under the curve; PPV, positive predictive value; NPV, negative predictive value.
