# Supplementary material for: ASAP Score versus GALAD Score for detection of hepatitis C-related hepatocellular carcinoma: A multicenter case-control analysis
Source: Front Oncol. 2022 Sep 30;12:1018396. doi: 10.3389/fonc.2022.1018396 (PMC9576185; doi:10.3389/fonc.2022.1018396)
Supplement: Supplementary file 1 [file Table_1.docx]

**Supplement Table 1.** Clinical characteristics of early-stage HCV-HCC

| N (%) | BCLC stage 0/A  (N=91) | 8^th^ TNM stage I  (N=92) |
| --- | --- | --- |
| Baseline characteristics |  |  |
| Age, years* | 60.0 ± 10.6 | 60.0 ± 10.6 |
| Male sex | 63 (69.2) | 66 (71.7) |
| Child-Pugh grade |  |  |
| A | 87 (95.6) | 89 (96.7) |
| B+C | 4 (4.4) | 3 (3.3) |
| Cirrhosis | 82 (90.1) | 81 (88.0) |
| Platelet, ×10^9^/L* | 137 (108, 152) | 142 (79, 187) |
| Bilirubin, μmol/L* | 16.9 (11.6, 26.5) | 11.8 (15.3, 21.7) |
| Albumin, g/L* | 44.2 ± 5.7 | 44.8 ± 5.8 |
| AFP, ng/mL* | 7.6 (2.8, 71.6) | 7.4 (2.8, 71.6) |
| Negative (< 20 ng/mL) | 42 (46.1) | 41 (44.6) |
| Positive (≥ 20 ng/mL) | 49 (53.9) | 51 (55.4) |
| PIVKA-II, mAU/ml* | 27.0 (19.8, 169.5) | 50.0 (27.7, 678.5) |
| Negative (< 40 mAU/mL) | 24 (26.4) | 22 (23.9) |
| Positive (≥ 40 mAU/mL) | 67 (73.6) | 70 (76.1) |
| AFP-L3, %* | 0.5 (2.0, 14.5) | 0.5 (2.0, 14.5) |
| Negative (< 10%) | 53 (58.2) | 51 (55.4) |
| Positive (≥ 10%) | 38 (41.8) | 41 (44.6) |
| Tumor characteristics |  |  |
| Tumor size, cm* | 4.1 (2.5, 7.1) | 4.1 (2.5, 7.1) |
| ≥ 3 cm | 44 (48.4) | 47 (51.1) |
| Multiple tumors | 19 (20.9) | 13 (14.1) |

*Values are mean ± standard deviation or median with interquartile range.

AFP, [alpha](app:ds:alpha)-fetoprotein; AFP-L3, lens culinaris agglutinin A-reactive fraction of alpha-fetoprotein; BCLC, Barcelona Clinic Liver Cancer; HCC, hepatocellular carcinoma; HCV, hepatitis C virus; PIVKA-II, protein induced by vitamin K absence or antagonist-II; TNM, tumor node metastasis
